# Postoperative bilateral visual loss after a single dose of tranexamic acid

**DOI:** 10.1002/anr3.70020

**Published:** 2025-07-09

**Authors:** B. Chevalley, M. Betello, E. Blavakis, A. Malclès, J. Maillard

**Affiliations:** ^1^ Division of Anaesthesiology, Department of Acute Care Medicine Geneva University Hospitals Geneva Switzerland; ^2^ Department of Ophthalmology Geneva University Hospitals Geneva Switzerland; ^3^ Faculty of Medicine University of Geneva, UNIGE Geneva Switzerland

**Keywords:** postoperative visual loss, tranexamic acid, visual disturbance

## Abstract

A 32‐year‐old woman presented with transient visual loss following the intra‐operative administration of a single intravenous dose of tranexamic acid during urgent cholecystectomy. Apart from obesity, the patient had no notable medical history or pre‐existing ocular conditions. Immediately after surgery, the patient reported sudden onset blindness. Ophthalmological and radiologic assessments did not reveal any detectable macrovascular or organic ophthalmic lesions. The patient's vision gradually improved spontaneously, with the resolution of the dyschromatopsia on the first postoperative day and complete recovery by the second postoperative day. This case highlights the importance of prompt investigation of acute visual impairment postoperatively and identifying potential causative agents, such as tranexamic acid.

## Introduction

Postoperative visual loss (POVL) is a rare but severe complication with an estimated incidence ranging from 1 in 60,000 to 1 in 125,000 anaesthetics [1]. Most cases are associated with spinal and cardiac surgery, often linked to factors, such as unusual head positioning, direct ocular pressure or thrombotic micro‐emboli. Surgery of the nose or paranasal sinuses is also associated with a higher risk of POVL [1]. The primary aetiologies include retinal artery occlusion, ischaemic optic neuropathy and more rarely stroke, transient ischaemic attack, acute glaucoma and corneal erosion. Non‐surgical risk factors for POVL include anaemia, the use of prolonged high‐dose vasopressors and the use of phosphodiesterase type 5 (PDE5) inhibitors.

Some cases of POVL have been associated with the administration of tranexamic acid (TXA). These have been associated with prolonged or repetitive use for treatment of metrorrhagia over several weeks; during major trauma surgery or in patients with pre‐existing thromboembolic risk [2, 3, 4]. We report a case of postoperative transient visual loss associated with dyschromatopsia potentially related to the intra‐operative administration of a single intravenous dose of TXA.

## Report

A 32‐year‐old woman presented to the emergency department with acute abdominal pain. She was diagnosed with cholelithiasis and underwent an urgent laparoscopic cholecystectomy. She was living with obesity (BMI 34 kg.m^−2^) and underwent an uncomplicated caesarean birth one month earlier. She had no documented bleeding disorder, abnormal laboratory results or any history of ophthalmological disease.

Before induction of anaesthesia, her observations were within normal limits: blood pressure 118/91 mmHg, heart rate 73 beats.min^−1^ and SpO_2_ 99% in air. Standard monitoring was applied, including 3‐lead ECG, SpO_2_ measurement, capnography, non‐invasive blood pressure monitoring and train‐of‐four monitoring. A rapid sequence induction was performed using propofol 220 mg, ketamine 25 mg, sufentanil 10 μg and succinylcholine 100 mg. The trachea was intubated uneventfully and anaesthesia was maintained with sevoflurane and intermittent sufentanil boluses (25 μg in total). Rocuronium was subsequently administered as intermittent boluses during pneumoperitoneum (total dose 70 mg).

In the context of ongoing low‐grade diffuse intra‐operative bleeding, despite meticulous surgical haemostasis, a single dose of TXA 1 g was administered intravenously, along with one unit of fresh frozen plasma (FFP). Simultaneously, invasive blood pressure monitoring via a radial arterial catheter was initiated as a precautionary step to facilitate monitoring and enable rapid blood sampling. No significant hypotensive episodes occurred during surgery. Vasopressor use was minimal, with a total of 600 μg of phenylephrine and 21 mg of ephedrine administered over 3 hours. Blood loss was less than 300 ml, with a haemoglobin nadir of 97 g.l^−1^, down from a baseline of 118 g.l^−1^. The patient received sugammadex 200 mg at the end of the procedure and the emergence from anaesthesia was uneventful.

Immediately after emergence, the patient reported bilateral painless vision loss. Ophthalmologic examination revealed visual acuity at ‘light perception’ in both eyes, semi‐midriatic pupils, lack of relative afferent pupillary defect and normal intra‐ocular pressure. Anterior segments and fundal exams were normal, indicating the absence of a lesion of the macula and the optic nerve. The neurological examination was otherwise normal. The remainder of the patient's clinical course, including surgical follow‐up, was uncomplicated.

An emergency cerebral computed tomography angiogram showed no signs of stroke or other vascular abnormalities. Cerebral magnetic resonance imaging showed no signs of ischaemia, posterior reversible encephalopathy syndrome (PRES) or other abnormalities. These investigations were performed on the day of onset of symptoms.

Daily ophthalmologic assessments revealed gradual spontaneous improvement from day 1 postoperatively, with normalised vision, but with residual dyschromatopsia. Visual function was completely normalised on day 2 postoperatively. The patient was discharged home three days after the surgery with a follow‐up three weeks later, which found the patient to have normal vision.

## Discussion

Tranexamic acid is a synthetic antifibrinolytic agent which binds to the lysine binding site on plasminogen. It inhibits plasmin formation which inhibits the degradation of the fibrin clot and promotes its stability [5]. Developed in the 1960s, this drug is currently used to prevent and treat bleeding in various contexts including menorrhagia, postpartum haemorrhage, major trauma and congenital or acquired coagulation disorders [5]. Used appropriately during major surgery, it can reduce the total amount of bleeding, the need for transfusions and the total amount of blood product transfused [6]. Tranexamic acid is generally well tolerated but significant side effects, such as permanent visual impairment, have been reported with its use [2].

Visual loss or disturbance associated with TXA is extremely rare. Most reported cases involve patients who received oral TXA over several weeks for metrorrhagia, with ophthalmologic evaluations revealing vascular damage, including central retinal artery occlusion and central retinal vein thrombosis [2, 3]. Transient blindness following repeated administration of TXA while undergoing long‐term dialysis has been reported after TXA was administered as part of the management of a peptic ulcer [7]. Another patient on dialysis developed nyctalopia following the administration of TXA, which was used to treat bleeding caused by duodenal angiodysplasia [8]. In patients with end‐stage renal failure on dialysis, a phenomenon of TXA accumulation can occur given it is almost exclusively renally eliminated. In these patients, sparing use of TXA is recommended, with a reduction in doses and frequency of administration [5].

Only one case of POVL directly associated with a single dose of TXA has been reported. It involved a young patient who sustained multiple injuries in a road traffic accident and subsequently underwent sequential orthopaedic and vascular surgical procedures for fractures of the lower limbs. Tranexamic acid was administered once during the third surgery in this sequence. Ophthalmologic and vascular assessments revealed no visible abnormalities and the condition gradually resolved following treatment with systemic corticosteroid, anticoagulant and antiplatelet agents [4].

There are two published case reports of postoperative dyschromatopsia without visual acuity reduction after administration of TXA. One case involved a child with factor VII deficiency who received multiple doses of TXA to prevent epistaxis and subsequently developed dyschromatopsia [9]. The other case of postoperative dyschromatopsia involved a patient who underwent two consecutive spinal neurosurgeries, during which multiple doses of TXA were administered [10].

Management strategies for POVL with or without dyschromatopsia depend on the underlying aetiology and may consist of systemic corticosteroids or intra‐ocular anti‐vascular endothelial growth factor injection [3].

The differential diagnosis for POVL includes macrovascular events, such as stroke or ocular artery thrombosis, PRES and intra‐operative ocular trauma. In this case, the potential causes for POVL and dyschromatopsia are very limited. General surgery is not known to increase the risk of visual loss.

We observed no significant haemodynamic instability which could contribute to this complication. The patient was supine with eyes properly closed and protected using adhesive eye patches. The patient did not receive any other medications which had been directly linked to POVL in the literature.

Estimated blood loss was less than 300 ml, although underestimation cannot be excluded. Aside from a moderate anaemia (97 g.l^−1^), no other laboratory abnormalities were observed. The patient underwent radiological and ophthalmic examinations on the same day the symptoms began. Notably, her caesarean birth was completed without notable complications, with less than 500 ml of blood loss. Postoperative thromboprophylaxis was managed with enoxaparin 40 mg once daily. Tranexamic acid 1 g was administered intravenously at that time without complications.

As most causes of postoperative blindness were excluded, the most likely explanation and possible risk factor identified in this case was the administration of TXA. This medication has been associated with POVL, either through classic ophthalmovascular lesions or by visual disturbances without any abnormal clinical findings. In the absence of another identifiable cause, the diagnosis of reversible postoperative blindness secondary to tranexamic acid was made. Although the pathophysiological mechanism is not clearly established, a direct pharmacodynamic effect of TXA on the retinal cone pigments involved in vision and retinal colour differentiation is a proposed hypothesis [10]. No treatment is necessary as this effect is transient and complete visual recovery is expected, but a close follow‐up of the patient is indicated in order to exclude other conditions.

It is important to note that the patient presented with certain risk factors at the time of care, including obesity and a caesarean birth one month earlier, during which a dose of TXA had been administered. The decision to administer TXA and to escalate monitoring, including arterial line placement, was made in a precautionary context, due to ongoing low‐volume bleeding despite prolonged surgical haemostasis and in anticipation of potential clinical or haemorrhagic deterioration. This approach of early or precautionary administration of TXA should be reconsidered as side effects, albeit rare, may be serious and disturbing to patients.

To our knowledge, this is the first documented case of POVL followed by postoperative dyschromatopsia secondary to intravenous TXA administration.

In order to detect potential problems promptly and to optimise care, patients treated with TXA long‐term should be instructed to seek medical attention immediately in the event of visual disturbances. These symptoms should be assessed by a specialist as soon as possible and discontinuation of treatment should be considered. This approach of actively searching for side effects should also be undertaken for patients treated in the acute phase, even with a limited number of administrations. Careful consideration should be given by anaesthetists to the potential side effects of TXA and unnecessary early administration avoided.
